# Anatomo‐Electro‐Clinical Features of Parietal Lobe Epilepsy: Insights From Scalp Video‐Electroencephalography

**DOI:** 10.1002/cns.70713

**Published:** 2026-01-09

**Authors:** Huijuan Wan, Xuemin Zhao, Wenhan Hu, Chao Zhang, Xiu Wang, Zhong Zheng, Shengsong Wang, Dandan Liu, Lin Sang, Xianghong Meng, Kai Zhang, Xiaoqiu Shao

**Affiliations:** ^1^ Department of Neurology and Department of Neuroscience, the First Affiliated Hospital of Xiamen University, School of Medicine Xiamen University Xiamen China; ^2^ Provincial Clinical Research Center for Brain Diseases Fujian China; ^3^ Department of Neurophysiology Beijing Neurosurgical Institute, Capital Medical University Beijing China; ^4^ Department of Neurosurgery Beijing Tiantan Hospital, Capital Medical University Beijing China; ^5^ Department of Neurosurgery Beijing Fengtai Hospital Beijing China; ^6^ Department of Neurology Beijing Tiantan Hospital, Capital Medical University Beijing China; ^7^ Department of Neurosurgery Shenzhen University General Hospital, Shenzhen University Shenzhen China

**Keywords:** ictal patterns, interictal patterns, refractory epilepsy, semiology

## Abstract

**Aims:**

To summarize the anatomo‐electro‐clinical characteristics of parietal lobe epilepsy (PLE) subgroups using unsupervised cluster analysis.

**Methods:**

This retrospective cohort study included patients with drug‐resistant PLE with seizure freedom after surgery and evaluated scalp video‐electroencephalography (EEG) recordings from three epilepsy centers. Hierarchical cluster analysis associated interictal/ictal patterns and initial ictal semiology with anatomical subgroups.

**Results:**

We analyzed 79 interictal EEG, 141 ictal EEG, and 141 semiological patterns in 47 patients. Cluster analysis associated interictal and ictal discharges from lateral superior parietal lobule epilepsy with centroparietal region distributions on scalp EEG, whereas discharges from other subgroups involved broader regions. Cluster heatmaps of the initial ictal semiology showed: Chapeau de gendarme, affective phenomena, and forced eye deviation in intraparietal sulcus; contralateral limb tonic/clonic or akinetic, affective phenomena, and visual illusions in SPL‐lateral; Chapeau de gendarme, behavioral arrest, and vestibular in parieto‐occipital sulcus; behavioral arrest in angular gyrus; distal gestural automatisms and cephalic sensations in posterior cingulate; body‐perception illusion and contralateral versive in supramarginal gyrus; contralateral facial tonic/clonic in parietal operculum.

**Conclusion:**

PLE subgroups exhibited distinct scalp EEG features and ictal semiology, reflecting unique propagation networks and highlighting the importance of detailed video‐EEG for identifying the epileptogenic zone and guiding intracranial electrode placement.

## Graphical abstract

Interictal and ictal discharges in lateral superior parietal lobule epilepsy are predominantly recorded in centroparietal region electrodes. The initial ictal semiology of different parietal lobe epilepsy subregions is distinct and specific, with minimal overlap, reflecting early propagation networks.

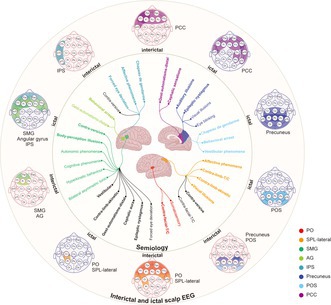

## 1 Introduction

Parietal lobe epilepsy (PLE) is a complex focal epilepsy with extensive synaptic networks and intricate connectivity to other brain regions [1, 2], which can contribute to misleading scalp electroencephalography (EEG) readings and clinical semiological features [3]. Owing to the difficulty in localizing the epileptogenic network, PLE surgery often has a lower success rate and less favorable prognosis than that of other lobe epilepsies [4]. Successful epilepsy surgery depends on establishing a solid anatomo‐electro‐clinical hypothesis during preoperative evaluation. Thus, accurate interpretation of semiology and scalp EEG is crucial for localizing the epileptogenic zone (EZ) and guiding intracranial electrode implantation, especially in magnetic resonance imaging (MRI)‐negative patients. PLE accounts for only 4%–6% of focal epilepsy cases [5, 6], and given the small sample size, research on PLE remains limited. The understanding of its distinct anatomo‐electro‐clinical features remains unclear, highlighting the need for larger studies with more participants.

Moreover, the definition of PLE varies across studies [3, 7, 8, 9]. Based on the functional integrity of the precentral and postcentral gyri and in accordance with the views of researchers such as Rasmussen [10] and Salanova [7, 9], we defined PLE as the EZ located in the posterior parietal cortex (region between the postcentral gyrus and occipital lobe), excluding the precentral and postcentral gyri, which are typically associated with central zone epilepsy. Unlike the relatively stereotypical nature of frontal lobe epilepsy, PLE tends to be more polymorphic in ictal semiology [11]. The objective symptoms that can be identified and the subjective symptoms remembered and reported by the patients not only reflect EZ manifestations but also the spread of discharges to other brain regions via different pathways. Therefore, analyzing each seizure type is essential, rather than focusing solely on habitual seizures reported by patients, along with the scalp EEG characteristics of the different PLE subgroups.

To address this, our study employed clustering techniques to analyze the semiology and scalp EEG discharge patterns associated with different sublobar regions of the parietal lobe. This study aimed to enhance our understanding of scalp video‐EEG information in PLE, reduce the risk of misinterpretation, and provide valuable insights for Phase I noninvasive preoperative assessment.

## 2 Methods

### 2.1 Patient Selection

**FIGURE 1:**
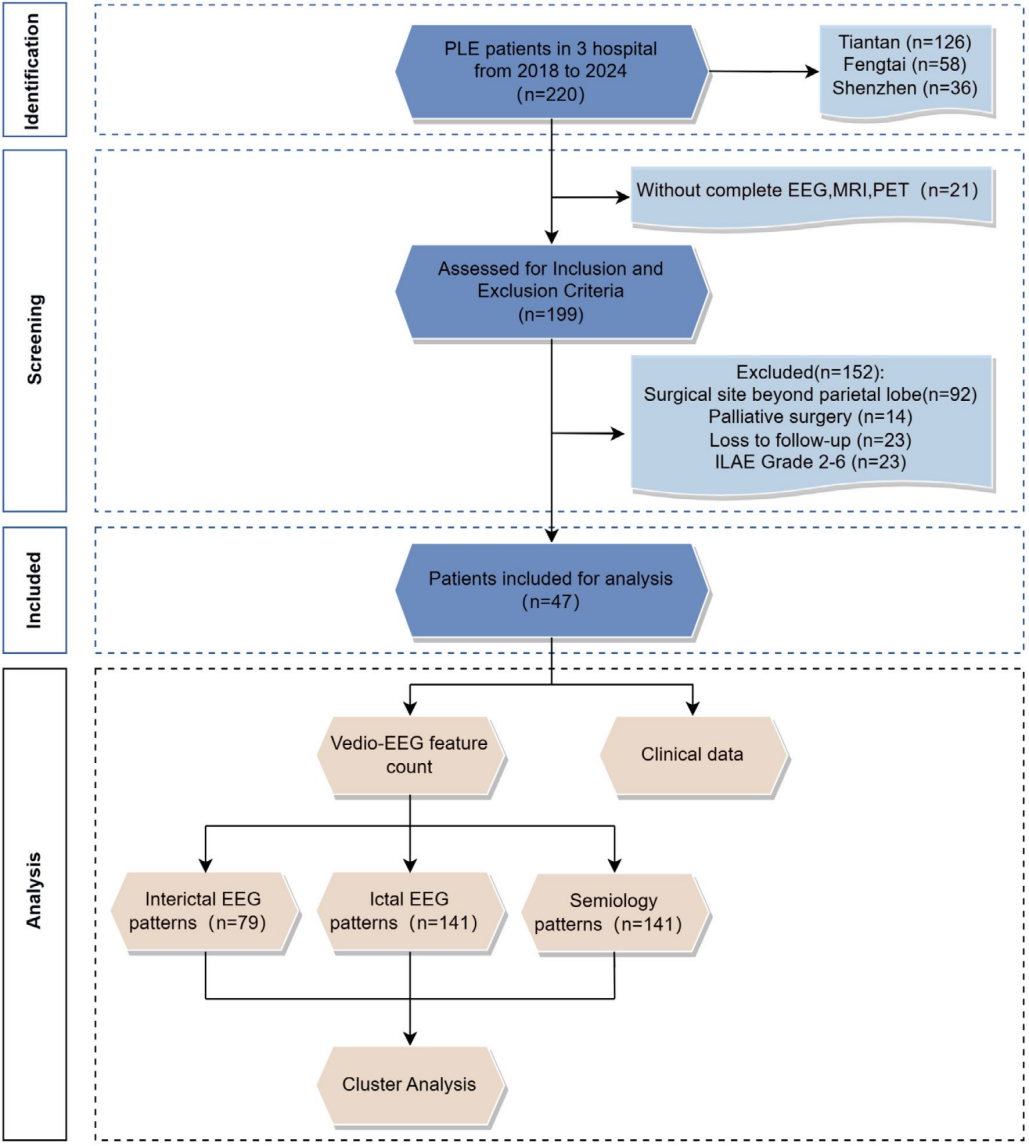
Flowchart. Flowchart depicting the identification process of 220 consecutive patients who underwent presurgical evaluations at three epilepsy centers and were diagnosed with parietal lobe epilepsy. This study included 79 scalp EEG interictal patterns, 141 scalp EEG ictal patterns, and 141 semiology patterns from 47 patients. EEG, electroencephalography; ILAE, International League Against Epilepsy; MRI, magnetic resonance imaging; PET, positron emission tomography.

We retrospectively selected 47 patients with drug‐resistant PLE who underwent surgery at the epilepsy centers of Beijing Tiantan Hospital, Beijing Fengtai Hospital, and Shenzhen University General Hospital between January 2018 and January 2024. The inclusion criteria were as follows: (1) completion of a comprehensive preoperative evaluation, including video‐EEG, 3.0 T epilepsy‐protocol MRI, and 2‐[18F] fluoro‐2‐deoxy‐d‐glucose (FDG) positron emission tomography (PET); stereoelectroencephalography (SEEG) was performed when necessary; all patients were reviewed by a multidisciplinary team to specify presurgical hypotheses based on the comprehensive examination results and determine the type of surgical intervention; (2) surgical intervention strictly confined to the parietal lobe; (3) surgical approach comprising resective surgery, laser interstitial thermal therapy (LITT), or radiofrequency thermocoagulation (RFTC); (4) achievement of seizure freedom (International League Against Epilepsy [ILAE] Class 1 [12]) with a minimum of 1 year of postoperative follow‐up. The exclusion criteria were as follows: (1) incomplete or unavailable follow‐up data, clinical information, video recordings for seizure semiology analysis, and scalp EEG data; (2) surgical intervention not limited to the parietal lobe. A flow diagram of study enrollment is shown in Figure 1.

### 2.2 Ethical Statement

This retrospective study was approved by the Ethics Committee of Beijing Tiantan Hospital (Approval No. KY2023‐034‐02), which served as the lead institutional review board for all participating centers, including Beijing Fengtai Hospital and Shenzhen University General Hospital. All procedures were conducted in accordance with the Declaration of Helsinki. Written informed consent for the use of clinical data was obtained from all patients at hospital admission, as required by the Ethics Committee of Beijing Tiantan Hospital.

### 2.3 Ictal and Interictal Scalp EEG Analysis

Scalp EEG data were independently reviewed by two experienced neurophysiologists (H.W. and S.W.) who were blinded to clinical information. Discrepancies were resolved through discussion or, if necessary, by a senior neurophysiologist (X.Q.). Each patient with PLE exhibited one or more interictal and ictal‐onset EEG patterns. To comprehensively characterize the EEG features of PLE, we analyzed the EEG data at two levels: individual EEG pattern and patient level. Each ictal EEG pattern was analyzed according to its morphology and distribution, whereas the interictal EEG was evaluated based solely on its distribution. Based on the literature [13, 14], the morphology of ictal onset was classified as low‐voltage fast activity (LVFA), spikes or sharp waves, obscured, and slow waves. EEG distribution was categorized as frontal, frontotemporal, temporal, centroparietal, and posterior. Further details of EEG analysis are provided in Text S1. Based on the relationship with the surgical intervention area, lateralization of the interictal discharges (IID) and ictal discharges (ID) was classified as ipsilateral to the surgical areas, contralateral, bilateral, or midline.

### 2.4 Semiology Analysis

With reference to the ILAE seizure semiology terminology [15, 16], two independent observers (H.W. and X.Z.) reviewed all seizure videos of each patient during EEG monitoring and recorded their semiological features. Discrepancies were resolved through discussion or, if necessary, by a senior neurophysiologist (X.Q.). We analyzed at least three seizures per patient, treating each seizure as a separate ictal semiology sample. For patients with more than three seizures, three different seizure types were selected for analysis to avoid overrepresentation of patients with multiple events. Given the complex semiology and dynamic propagation pathways in PLE, our analysis focused exclusively on the initial ictal semiology (the first semiological features) of each seizure to minimize confounding from secondary spread.

### 2.5 EZ Definition and Regional Delineation

Determination of the EZ was supported by the sustained seizure‐free outcome observed postoperatively, providing evidence that the EZ was accurately localized and effectively targeted during preoperative planning [17]. For patients who underwent SEEG, the seizure onset zone identified via SEEG was incorporated into EZ localization. For cases without SEEG, the surgical intervention site served as a proxy for the EZ, encompassing the seizure “generator” and early semiology [18].

For semiology cluster analysis, the brain regions involved in the surgical intervention were divided into eight subgroups: lateral superior parietal lobule (SPL‐lateral), precuneus, angular gyrus (AG), supramarginal gyrus (SMG), posterior cingulate cortex (PCC), parieto‐occipital sulcus (POS), intraparietal sulcus (IPS), and parietal operculum (PO). The cortical location of the surgical intervention was determined based on registration of postoperative thin‐slice computed tomography scans with preoperative MRI images and compared with the preoperative hypothesis. For certain patients, the surgical boundaries extended beyond a single subregion. Given the limitations of scalp EEG's spatial resolution, these patients were classified as “multisublobar” in the EEG cluster analysis.

### 2.6 Accurate Localization and Lateralization

Accurate localization was defined when findings from noninvasive preoperative evaluations (EEG, MRI, seizure semiology, and PET) concordantly identified the same region as the surgical target. Accurate lateralization was determined when these findings were concordant at the hemispheric level.

### 2.7 Statistical Analyses

Statistical analyses were performed using R v. 4.1.2 and Python v.3.6.10. Normality of data distribution was assessed using the Shapiro–Wilk test. Descriptive analyses summarized the clinical characteristics of the patients and measured the accuracy of each noninvasive preoperative assessment (scalp EEG, MRI [18], FDG‐PET). We performed an unsupervised agglomerative hierarchical cluster analysis on the ictal onset pattern (distribution and morphology) of the scalp EEG recordings and involved brain areas, as well as the IID distribution of scalp EEG and involved brain areas. By iteratively merging the closest cluster pairs, nodes were combined into larger clusters to summarize scalp EEG patterns across different brain areas involved. The average linkage method was used as the clustering criterion, and Pearson's correlation was used to measure the distance (or similarity) between clusters. A hierarchical nested dendrogram was created by calculating the similarity between data points from different categories to identify closely related clusters and distinct subgroups. Given the spatial resolution limitations of scalp EEG, patients whose surgical boundaries crossed a single parietal subregion were classified as “multisublobar” in the scalp EEG clustering analysis, in order to minimize the impact of multisublobar cases on the EEG clustering results. To evaluate the impact of varying cluster numbers on clustering performance, three standard clustering validity indices were utilized: Silhouette Score, Calinski–Harabasz Index, and Davies–Bouldin Index. For visualization, dimensionality reduction was conducted using Uniform Manifold Approximation and Projection (UMAP) and t‐distributed stochastic neighbor embedding (t‐SNE), projecting high‐dimensional data into a two‐dimensional space.

Additionally, hierarchical clustering was performed separately for involved brain areas (brain areas × patients) and initial ictal semiological components (semiology × patients), resulting in a rearranged series of symptoms and brain areas based on similarity. Subsequently, Pearson's correlation was used to calculate the strength of the association between involved brain areas and initial ictal semiology. Significance was assessed, and correlations with *p* < 0.05 and *p* < 0.1 were marked in the clustered heatmap for visualization. For cases in which the surgical intervention involved multiple brain areas, each area was independently assigned a value of 1 in the brain area–symptom cluster analysis to ensure that the contribution of all relevant areas was adequately represented. Sex was not included as a clustering variable, as supplementary analyses showed no significant effect on EEG patterns, semiology, or involved parietal subgroups (Text S4 and Tables S1–S4).

## 3 Results

### 3.1 Patient Characteristics

The 47 patients with PLE included 33 (70.2%) men and 14 (29.8%) women, with a mean age at surgery of 23.3 ± 9.9 years. Cerebral MRI results were negative in 12 (25.5%) patients. Of the 47 patients, 43 (91.5%) underwent resective surgery, two (4.3%) underwent LITT, and two (4.3%) underwent RFTC. Across the whole group, ictal EEG, interictal EEG, semiology, MRI, and PET correctly localized the EZ in 36.2%, 14.9%, 34%, 63.8%, and 66% of patients, respectively. Additional patient characteristics are presented in Table 1. Twenty‐eight patients (59.6%) underwent SEEG. The clinical, demographic, sampling regions, and scalp EEG onset patterns of SEEG‐implanted patients are presented in Table S5.

**TABLE 1:** Characteristics of patients with PLE.

Characteristic	PLE *N* = 47
Sex, *n*, women/men (% women)	14/33 (29.8)
Age at onset, y, median (IQR)	10 (6–13)
Age at surgery, y, mean (SD)	23.3 (9.9)
History of FBTCS, *n* (%)	37 (78.7)
Preoperative seizure frequency, *n* (%)	
> 10/day	5 (10.6)
1/Daily	19 (40.4)
Weekly	11 (23.4)
Monthly	12 (25.5)
SEEG recording, *n* (%)	28 (59.6)
Surgery	
Surgery location, *n* (%)	
Single sublobar	
SPL‐lateral	7 (14.9)
SMG	18 (38.2)
AG	1 (2.1)
POS	2 (4.3)
IPS	3 (6.4)
PCC	4 (8.5)
PO	2 (4.3)
Precuneus	3 (6.4)
Multisublobar	7 (14.9)
SPL‐lateral, SMG	2 (4.3)
SPL‐lateral, precuneus, PCC, SMG	1 (2.1)
SPL‐lateral, precuneus	1 (2.1)
SPL‐lateral, precuneus, PCC	1 (2.1)
SMG, PO	1 (2.1)
SPL‐lateral, AG, POS, IPS	1 (2.1)
Surgery type, *n* (%)	
Resective	43 (91.5)
LITT	2 (4.3)
RFTC	2 (4.3)
Follow‐up time after surgery, m, median (IQR)	24 (13–36)
Seizure number, times, median (IQR)	4 (3–6)
EEG recording time, d, median (IQR)	3 (2–5)
Histopathological types, *n* (%)^a^	
Gangliocytoma	1 (2.3)
Glioma	1 (2.3)
FCD	28 (65.1)
Nonspecific	3 (7)
Gliosis	6 (14)
Gyral scarring	2 (4.7)
DNET	1 (2.3)
Cavernous hemangioma	1 (2.3)
Accurate localization, *n* (%)	
Ictal EEG	17 (36.2)
Interictal EEG	7 (14.9)
Semiology	16 (34)
MRI	30 (63.8)
PET	31 (66)
Accurate lateralization, *n* (%)	
Ictal EEG	37 (78.7)
Interictal EEG	27 (57.4)
Semiology	33 (70.2)
MRI	32 (68.1)
PET	39 (83)

Abbreviations: AG, angular gyrus; DNET, dysembryoplastic neuroepithelial tumor; EEG, electroencephalography; FBTCS, focal to bilateral tonic–clonic seizures; FCD, focal cortical dysplasia; IPL, inferior parietal lobule; IPS, intraparietal sulcus; IQR, interquartile range; LITT, laser interstitial thermal therapy; MRI, magnetic resonance imaging; PCC, posterior cingulate cortex; PET, positron emission tomography; PLE, parietal lobe epilepsy; PO, parietal operculum; POS, parieto‐occipital sulcus; RFTC, radiofrequency thermocoagulation; SMG, supramarginal gyrus; SPL, superior parietal lobule.

^a^ Percentages for histopathological types are based on patients with available pathological data (*n* = 43), as data were missing for four patients from the full cohort (*N* = 47).

### 3.2 Scalp EEG Characteristics

**FIGURE 2:**
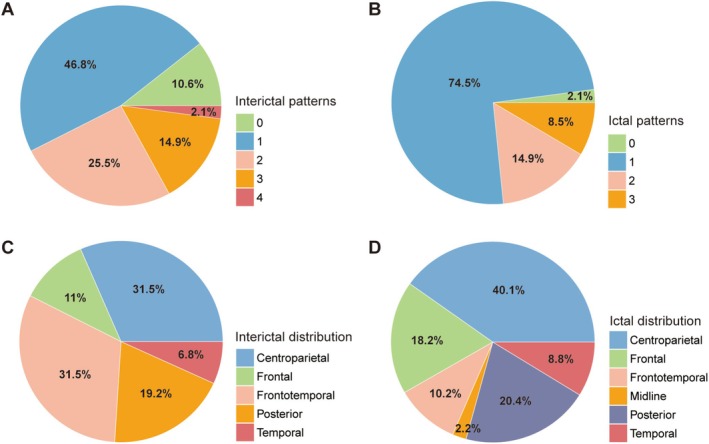
Proportion and Distribution of Interictal and Ictal Scalp EEG Patterns in Patients with PLE. (A) Pie chart representing the proportion of interictal scalp EEG patterns in patients with PLE. (B) Pie chart representing the proportion of ictal scalp EEG patterns in patients with PLE. (C) Pie chart representing the distribution of interictal scalp EEG. (D) Pie chart representing the distribution of ictal scalp EEG. EEG, electroencephalography; PLE, parietal lobe epilepsy.

In total, 304 seizures were recorded and analyzed via EEG, with each patient having between 3 and 22 seizures (median: 4). Multiple EEG patterns were observed in 42.5% of patients during the interictal period, and 23.4% also showed multiple patterns during the ictal period (Figure 2A,B). In subsequent analyses, 79 interictal and 141 ictal scalp EEG patterns were included. IID and ID were broadly distributed across multiple brain regions. Figure S1 shows that, among ictal morphologies, LVFA achieved the highest accuracy in localization (61.9%) and lateralization (93.7%).

### 3.3 Scalp EEG Features Across PLE Subgroups

The final number of clusters was selected by integrating quantitative clustering metrics with qualitative assessment of UMAP and t‐SNE visualizations (Figures S2 and S3 and Text S2).

**FIGURE 3:**
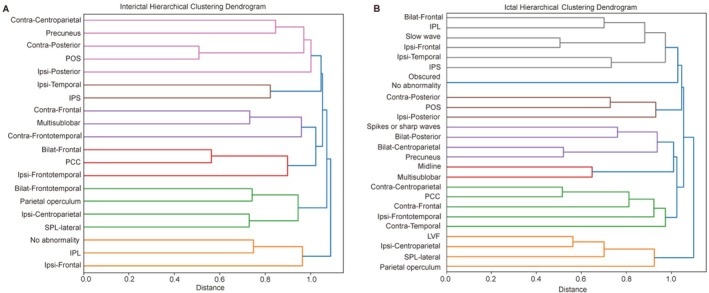
Dendrogram Based on Hierarchical Cluster Analysis of Interictal (A) and Ictal (B) Scalp EEG Patterns in Patients with PLE. The horizontal axis represents the linkage distance, derived from Pearson's correlation coefficients and computed using the average linkage method. Bilat, bilateral; Contra, contralateral; EEG, electroencephalography; IPL, inferior parietal lobule; IPS, intraparietal sulcus; Ipsi, ipsilateral; PCC, posterior cingulate cortex; PLE, parietal lobe epilepsy; POS, parieto‐occipital sulcus; SPL, superior parietal lobule.

**FIGURE 4:**
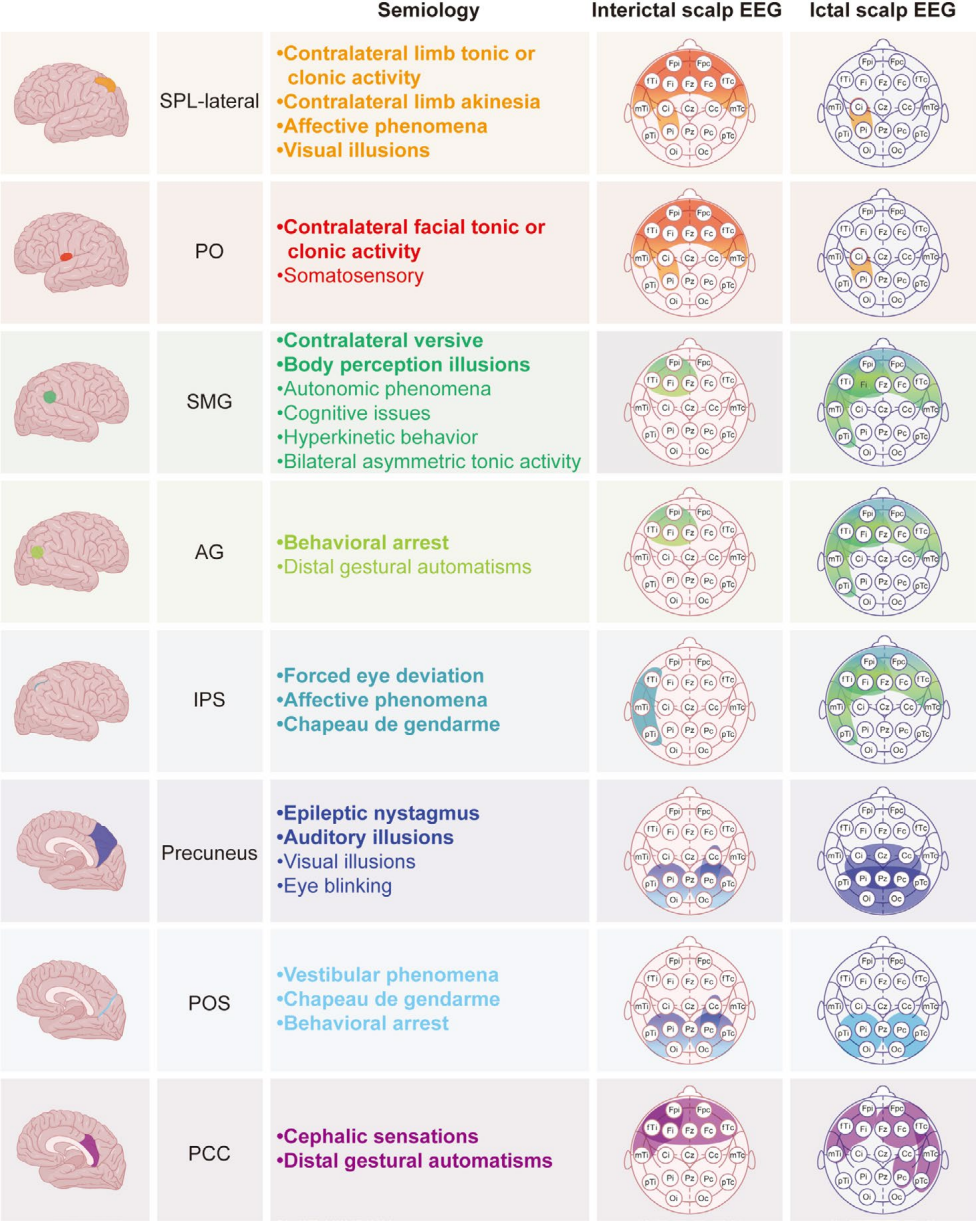
Integrated Summary of Study Results. The eight parietal lobe subgroups are represented by distinct colors. In the Integrated Summary Figure, the semiology section only displays the positive symptoms (i.e., those positively associated with each subgroup) shown in Figure 5. Bold font indicates statistical significance (*p* < 0.05), while regular font represents trend‐level associations (*p* < 0.1). The electrodes involved in the ictal and interictal discharges are circled in the representative color of their corresponding subgroups. When two subgroups have similar discharge distributions, a color gradient between their representative colors is used to visually indicate this relationship. Electrode names follow the international 10–20 system. To enhance clarity, the left and right hemispheres are not distinguished, and electrodes are grouped according to surgical laterality: Ipsilateral (i) and contralateral (c) to the intervention side. The electrodes F7/F8, T3/T4, and T5/T6 have been renamed as anterior temporal (fTi and fTc), middle temporal (mTi and mTc), and posterior temporal (pTi and pTc). Here, “f” stands for frontal, “m” for middle, and “p” for posterior. AG, angular gyrus; Contra, contralateral; EEG, electroencephalography; EZ, epileptogenic zone; Gest, gestural; IPS, intraparietal sulcus; Ipsi, ipsilateral; PCC, posterior cingulate cortex; PO, parietal operculum; POS, parieto‐occipital sulcus; SMG, supramarginal gyrus; SPL, superior parietal lobule; T/C, tonic/clonic.

Hierarchical cluster analysis of the IID distribution in the scalp EEG and involved brain areas from the 79 analyzed interictal EEG patterns identified six major clusters (Figures 3A and 4; Text S3). These clusters differentiated region‐specific interictal patterns among PLE subgroups. Notably, in the fifth cluster, smaller sub‐clusters at a lower level grouped the IID on the centroparietal region electrodes and SPL‐lateral together, separating them from the PO and IID on the bilateral frontal‐temporal region electrodes. Similarly, hierarchical cluster analysis of the ictal onset patterns in the scalp EEG and involved brain areas from 141 seizures identified seven major clusters (Figures 3B and 4; Text S3), revealing region‐specific ictal signatures among PLE subgroups. Smaller sub‐clusters at a lower level in the sixth cluster grouped the ID on the centroparietal region electrodes, LVFA onset, and SPL‐lateral together, separating them from the PO.

### 3.4 Semiology Characteristics

**FIGURE 5:**
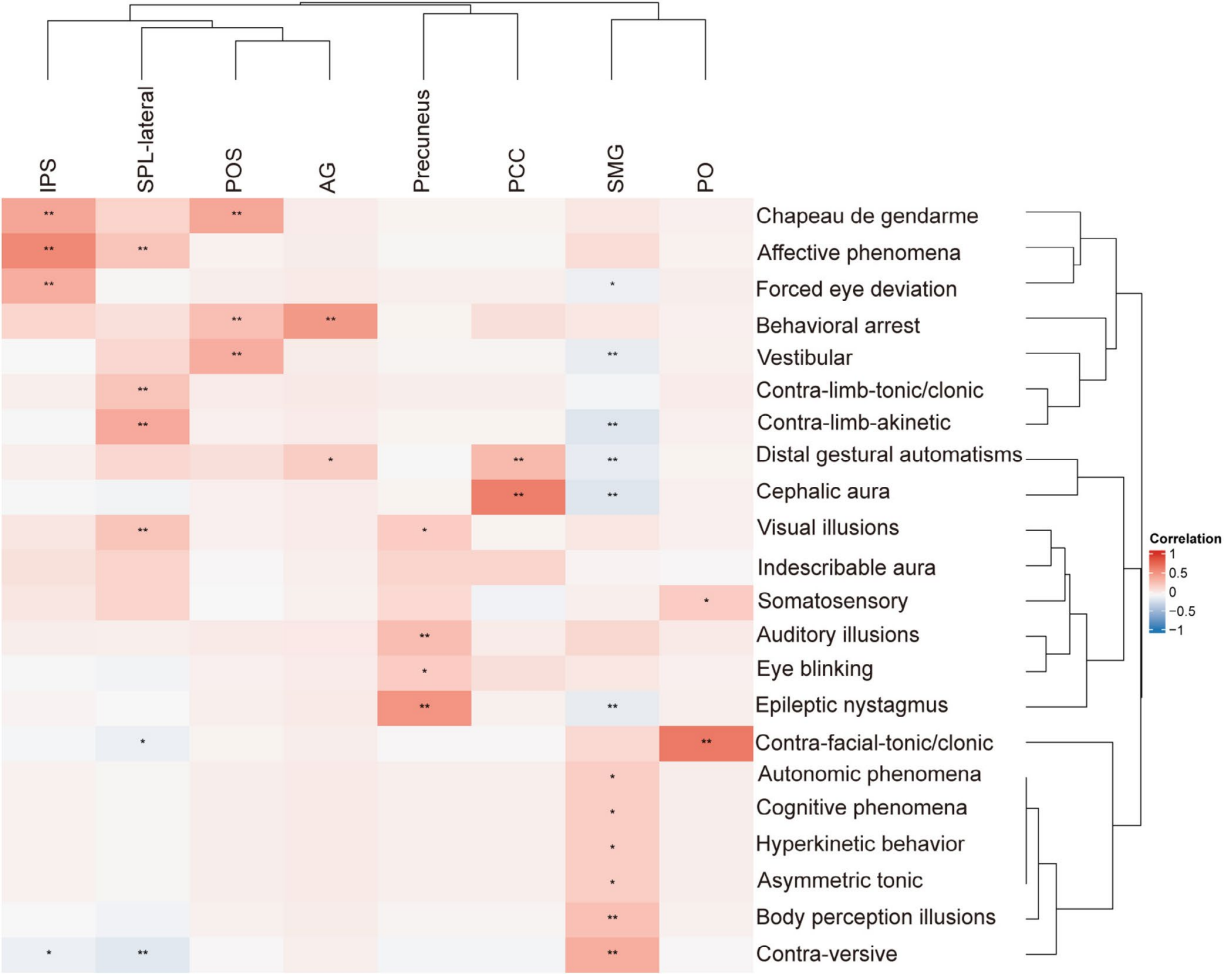
Cluster Heatmap of Pearson's Correlation and Hierarchical Clustering Analyses Between Subgroups and Initial Ictal Semiology with Significant Differences. The horizontal axis represents different brain subgroups, and the vertical axis represents various initial ictal semiological features. The center of the figure displays a Pearson's correlation‐based clustering heatmap, where color intensity reflects the strength and direction of the correlation: Red for positive and blue for negative associations. Both rows and columns were hierarchically clustered to highlight patterns of association. Asterisks denote statistical significance: * *p* < 0.1 and **p < 0.05. AG, angular gyrus; Contra, contralateral; IPL, inferior parietal lobule; IPS, intraparietal sulcus; PCC, posterior cingulate cortex; PO, parietal operculum; POS, parieto‐occipital sulcus; SMG, supramarginal gyrus; SPL, superior parietal lobule.

In total, 22 ictal semiological components (initial ictal semiology of each seizure) were recorded across all the included seizures (Figures 4 and 5). Of these, 11 (50%) components were classified as not observable or possibly observable manifestations, corresponding to what has previously been referred to as “aura” in the literature. At the patient level, 26 (55.3%) patients reported nonobservable or possibly observable manifestations as the initial symptoms of their seizures, and five (10.6%) experienced two distinct types of such manifestations. At the seizure level, somatosensory symptoms were the most frequently reported initial symptom (12.8%), with other manifestations detailed in Figure S4.

### 3.5 Semiology Features Across PLE Subgroups

The initial ictal semiological components and involved brain areas, classified using hierarchical cluster analysis, along with their correlations, are shown in Figure 4 (all patients), Figure S5 (SEEG patients), and Figure 5. A positive correlation indicates that a specific symptom is consistently associated with certain brain areas, whereas a negative correlation indicates that a symptom is never associated with those brain areas.

## 4 Discussion

In this study, we analyzed 79 scalp EEG interictal, 141 scalp EEG ictal, and 141 semiology patterns from 47 patients with pure PLE—the largest series reported to date. Our findings suggest that interictal and ictal EEG discharges in PLE are often distributed outside the involved brain areas, even appearing in contralateral or bilateral regions. Notably, when the EZ is located in the SPL‐lateral, scalp EEG frequently provides accurate localization to the ipsilateral centroparietal electrodes during the interictal and ictal periods. Additionally, initial ictal semiology varies across different PLE subgroups with minimal overlap, potentially reflecting distinct early propagation networks.

Our findings support the long‐standing view that no specific scalp EEG pattern can reliably pinpoint the exact intracranial seizure origin [13]. This limitation arises from the biophysical nature of EEG signal propagation: electric fields generated by synchronized neuronal activity are recorded on the scalp surface via volume conduction [19]. However, owing to the interposition of cerebrospinal fluid, meninges, skull, and subcutaneous tissue between the brain generators and scalp electrodes, these signals undergo substantial spatial dispersion and amplitude attenuation, compromising the spatial resolution of scalp EEG [19, 20]. Moreover, deep or multifocal seizure origins, source connectivity, and rapid ictal propagation further reduce localization accuracy. This instability is particularly pronounced in PLE, where studies have reported a higher likelihood of interictal and ictal EEG discharges being falsely localized to other lobes compared to frontal or temporal lobe epilepsy [3]. Our study found that 42.5% and 23.4% of patients exhibited multiple interictal and multiple ictal EEG patterns, respectively. Only 29.1% of IID and 39.0% of ID were accurately localized to the centroparietal electrode, with ictal and interictal localization achieved in 36.2% and 14.9% of cases, respectively. These proportions are comparable to those reported previously [9, 21]. Despite these limitations, EEG remains essential for presurgical evaluation, especially when imaging fails to identify epileptogenic abnormalities. EEG is also crucial for guiding further diagnostic workups and surgical decisions. Given the heterogeneity of EEG patterns in PLE, our clustering analysis offers a data‐driven approach to identify distinct phenotypic subgroups with divergent EEG patterns and corresponding EZ distributions.

Although PLE generally shows widespread interictal EEG activity, ID can rapidly propagate across cortical areas [3]. Our findings provide a novel insight, indicating that different subgroups of the parietal lobe exhibit distinct patterns of scalp EEG distribution.

Specifically, scalp EEG signals from the EZ in the SPL‐lateral region demonstrated relatively high accuracy in localizing the EZ during interictal and ictal periods. For the EZ in the PO, ictal scalp EEG localization is generally reliable; however, IID beyond the ipsilateral centroparietal region may also involve the bilateral frontotemporal regions. For the EZ in the PCC and precuneus, the distribution of scalp EEG activity does not fully align with the EZ, as discharges originating from these deep cortical regions may propagate to other scalp areas or even to the contralateral hemisphere. For the EZ in the POS, both IID and ID were predominantly distributed over ipsilateral or bilateral posterior regions. For the EZ in the IPS, IID and ID were most often observed in the ipsilateral temporal region, although some ID may have extended to the ipsilateral or bilateral frontal regions. For the EZ in the IPL, IID may be distributed in the ipsilateral frontal region or may be absent altogether, whereas ID is more frequently observed in the ipsilateral or bilateral frontal regions and may also appear in the ipsilateral temporal region. From an anatomical and electrophysiological perspective, these findings can be interpreted as follows: the SPL‐lateral is located closer to the cortical surface, making the electric fields generated by its neuronal discharges more likely to penetrate the meninges and skull and be accurately captured by scalp electrodes [22]. In contrast, the precuneus and PCC, situated medially or in deeper cortical layers, tend to experience signal attenuation and scattering during propagation and may project to incorrect scalp locations. Moreover, neuronal activity in the POS and IPS can propagate over long distances but may undergo partial cancelation because of the opposing discharge orientations of pyramidal neuron populations on opposite sulcal walls, potentially compromising the accuracy of scalp EEG spatial localization [22]. Parietal lobe seizures can rapidly propagate to other lobes or the contralateral medial parietal cortex via major white matter pathways, such as the superior longitudinal fasciculus, middle longitudinal fasciculus, arcuate fasciculus, and corpus callosum, contributing to the diffuse distribution of parietal EEG activity on the scalp [1]. Among parietal lobe subgroups, the SPL‐lateral, with the least involvement in these major white matter pathways [1, 23], may account for its superior spatial localization accuracy in scalp EEG.

Moreover, our study found that SPL‐lateral seizures are associated with LVFA, which shows the highest localization accuracy among seizure onset patterns [24]. Taken together, these findings highlight the importance of considering specific cortical regions when interpreting EEG patterns in PLE. Careful analysis of scalp EEG features can provide valuable clues for EZ localization. In patients with complex or widespread discharge patterns, the possibility of PLE should not be overlooked.

Consistent with previous literature, our data demonstrated significant intrapatient heterogeneity in seizure semiology among individuals with PLE, where a single patient may exhibit multiple seizure types and initial ictal semiology, including nonobservable or possibly observable manifestations, as defined in the ILAE 2025 classification [1, 9, 11, 18, 25]. This supports the hypothesis of multifocal propagation pathways owing to the high connectivity of the parietal lobe [11]. During presurgical evaluation, this multidirectional propagation characteristic may produce seizure symptoms that mimic activity in remote brain regions, obscuring the true EZ and increasing localization errors. Nonobservable or possibly observable manifestations, which are subjective and occur at seizure onset, can provide crucial clues for EZ localization [26]. However, their usefulness in PLE is limited by several factors: (1) seizures originating from the noneloquent parietal cortex may not produce identifiable manifestations [7]; (2) these subjective manifestations may be absent if consciousness or memory is impaired [27]; and (3) the reliability of subjective reports is influenced by the age, cognitive function, and emotional state of the patients and observers [26]. In our cohort, 55.3% of patients reported nonobservable or possibly observable manifestations as the initial symptoms, consistent with prior studies [7]. Notably, 10.6% of the patients experienced two distinct types of these subjective manifestations at seizure onset, further complicating EZ interpretation. For patients without clear subjective manifestations, the first objectively identifiable symptom (e.g., motor manifestations) may provide equally important localization information. As a higher‐order association cortex, the parietal lobe mediates functions such as spatial awareness and sensory integration, often without direct correspondence to sensory or motor symptoms. Consequently, in some patients with PLE, the EZ may be distant from the seizure onset zone, with clinically recognizable symptoms emerging only when epileptic discharges propagate beyond the parietal association areas to eloquent cortical regions [7]. To address these challenges, we systematically analyzed the initial symptoms of each seizure episode—whether or not they included observable manifestations—to identify early propagation patterns in PLE and their association with specific parietal subgroups.

In the 19 cases without SEEG, the EZ identified based on the surgical intervention site may be overestimated (potentially including early propagation areas). According to Table S6, 18 patients had surgical interventions limited to a single subregion, thereby aligning with our subgroup hierarchy classification. We believe this situation reduces the potential interference that could affect the accuracy of clustering analysis due to the inclusion of early propagation areas in the surgical intervention site. Furthermore, we did not delineate precise electrical propagation pathways based on SEEG electrode sequences. However, our study aimed to provide clues for EZ localization through noninvasive examinations, guiding intracranial electrode placement or surgical intervention. Our results support the view that PLE has a diverse spreading network, meaning cluster analysis cannot define a fixed symptom pattern for specific subgroups. Cluster analysis of initial seizure symptoms and parietal lobe subgroups showed that early symptoms in different subgroups were distinct with minimal overlap, suggesting that PLE follows a complex, nonrandom pattern that partially reflects early propagation networks.

Initial symptoms associated with the EZ in the IPS include forced eye deviation, which may indicate involvement of the parietal eye area on the posterior part of the medial wall of the IPS [28], or propagation through the rostral IPS to the premotor and prefrontal areas, causing frontal eye field involvement [1]. Affective phenomena and Chapeau de gendarme suggest early spread to the anterior prefrontal and limbic systems [17, 29]. Initial symptoms associated with the EZ in the SPL‐lateral include contralateral limb tonic or clonic activity, contralateral limb akinetic, affective phenomena, and visual illusions, reflecting activation of the primary motor cortex, premotor cortex, and occipital lobe activation [30]. The EZ in the AG showed complex and often subtle semiology, evolving into behavioral arrest and distal gestural automatisms following rapid propagation to temporal‐limbic regions [31, 32]. EZ in the POS typically presented with nonspecific symptoms, such as vestibular phenomena, consistent with its overlap with parietal, temporal, occipital, and insular networks; however, Chapeau de gendarme and behavioral arrest suggest further spread to the frontal or temporal lobes. EZ in the precuneus may initially present with visual illusions and eye blinking, supported by previous electrical stimulation studies [30]. Epileptic nystagmus and auditory illusions in these cases suggest propagation to the occipital or temporal lobes [33, 34]. EZ in the PCC usually presents as paucisymptomatic [35] but may produce distal gestural automatism, likely because of spread to mesial temporal structures via hippocampal connectivity [36], mimicking mesial temporal lobe epilepsy [35]. Cephalic sensations, although considered nonspecific and previously attributed to temporal structures such as the amygdala and entorhinal cortex [27], were also observed, supporting the propagation from the PCC to the temporal lobe. Several initial semiological features were linked to the EZ in the SMG. Body perception illusions were frequently observed possibly resulting from disruption of the normal body representation integration [37]. Contralateral versive likely reflects close functional fiber connections between the SMG and frontal lobe [23]. Autonomic phenomena, cognitive phenomena, hyperkinetic behavior, and bilateral asymmetric tonic posturing showed marginal associations with the SMG, suggesting possible propagation to frontal or temporal regions. Symptoms associated with the EZ in the PO include contralateral facial tonic or clonic activity, indicating involvement of the facial representation area in the frontocentral operculum, located anteriorly in the PO [38], and somatosensory manifestations, reflecting the PO's role in the somatosensory processing network [38, 39].

This study had some limitations. First, the sample size was relatively small, primarily because PLE often presents with subtle clinical manifestations and complex propagation pathways. To ensure the specificity of the study population and accuracy of our conclusions, we strictly included only cases of “pure PLE,” excluding patients whose EZs extended into adjacent lobes, minimizing confounding from extraparietal propagation. Second, the sample size for some subgroups was insufficient, leading to an uneven distribution of cases, which could impact statistical power and reliability of the findings. Further validation in larger cohorts with more evenly distributed cases is warranted to enhance the statistical robustness of the results. Third, in cases without SEEG, we cannot fully exclude the possibility that the EZ includes early propagation areas. To mitigate this, we repeated our main analysis exclusively for patients who underwent SEEG, and the clustering results (Figure S5) showed no significant differences. We did not limit our cohort to only patients who underwent SEEG to provide the most comprehensive semiological description of PLE.

## 5 Conclusions

This study systematically analyzed the anatomo‐electro‐clinical features of PLE, highlighting the importance of thoroughly evaluating EEG patterns and initial semiological features using scalp EEG during Phase I presurgical assessment to improve preoperative hypotheses or guide intracranial electrode placement.

## Author Contributions

Conception and design of the study: Xiaoqiu Shao, Kai Zhang, Huijuan Wan; Data acquisition and analysis: Huijuan Wan, Xuemin Zhao, Wenhan Hu, Chao Zhang, Xiu Wang, Shengsong Wang, Dandan Liu, Xiaoqiu Shao; Interpretation of data: Dandan Liu, Zhong Zheng, Lin Sang, Xianghong Meng, Kai Zhang, Xiaoqiu Shao; Drafting and critically revising the article: Huijuan Wan, Xuemin Zhao; Critically revising the article with respect to intellectual content: Kai Zhang, Lin Sang, Xianghong Meng, Chao Zhang, Xiu Wang, Wenhan Hu, Xiaoqiu Shao; All authors read and approved the final manuscript.

## Funding

This research was funded by the Xiamen High‐Quality Development Program for Young and Middle‐Aged Key Professionals (Grant No. 2024GZL‐GG80) and the Natural Science Foundation of Xiamen City (Grant No. 3502Z20227092).

## Disclosure

The authors have nothing to report.

## Ethics Statement

We confirm that we have read the journal's position on ethical publication and affirm that this report is consistent with those guidelines.

## Conflicts of Interest

The authors declare no conflicts of interest.

## Supporting information


**Figure S1:** Proportion of correct localization and lateralization for different morphological types of ictal scalp electroencephalography (EEG) in patients with parietal lobe epilepsy (PLE).

**Figure S2:** Clustering Validation and Selection of Optimal Cluster Number for Interictal EEG Patterns.

**Figure S3:** Clustering Validation and Selection of Optimal Cluster Number for Ictal EEG Patterns.

**Figure S4:** Characteristics of not observable or possibly observable manifestations.

**Figure S5:** Cluster Heatmap of Pearson's Correlation and Hierarchical Clustering Analyses Between Subgroups and Initial Ictal Semiology with Significant Differences in Patients Undergoing SEEG.

**Data S1:** cns70713‐sup‐0006‐DataS1.docx.

**Table S1:** Comparison of epileptogenic parietal subgroups between men and women patients with PLE.
**Table S2:** Comparison of EEG interictal distribution between men and women patients with PLE.
**Table S3:** Comparison of EEG ictal patterns between men and women patients with PLE.
**Table S4:** Comparison of initial ictal semiology between men and women patients with PLE.
**Table S5:** SEEG‐Implanted Patients: Clinical, Sampling, and Scalp EEG Onset Data (*n* = 28).
**Table S6:** Clinical and Demographic Data for Non‐SEEG Patients (*n* = 19).

**Data S2:** cns70713‐sup‐0008‐DataS2.docx.

## Data Availability

The data that support the findings of this study are available on request from the corresponding author. The data are not publicly available due to privacy or ethical restrictions.
