# Supplementary figures and images for: Anatomo‐Electro‐Clinical Features of Parietal Lobe Epilepsy: Insights From Scalp Video‐Electroencephalography
Source: CNS Neurosci Ther. 2026 Jan 9;32(1):e70713. doi: 10.1002/cns.70713 (PMC12784376; doi:10.1002/cns.70713)

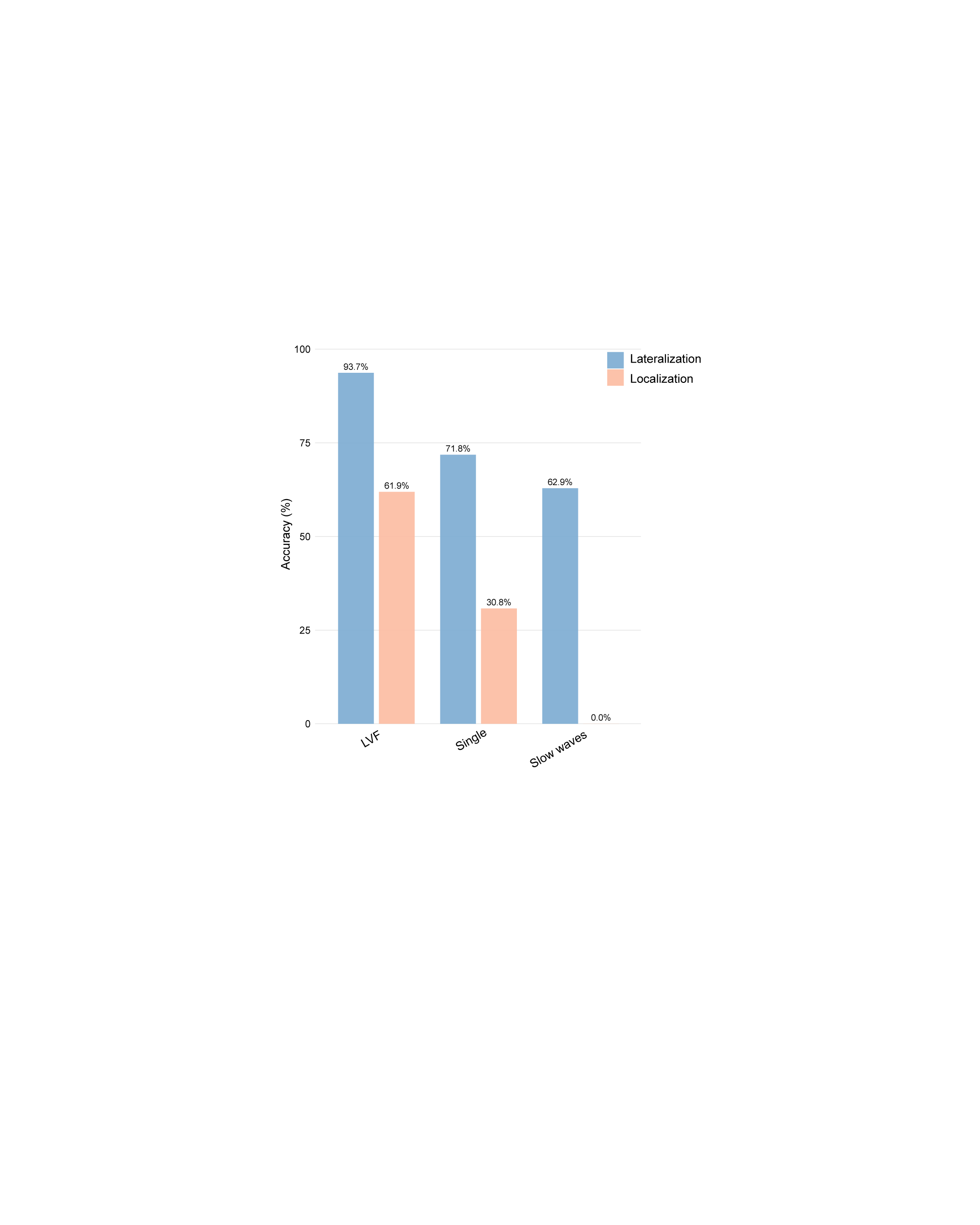

Supplement: Supplementary file 1 — Figure S1: Proportion of correct localization and lateralization for different morphological types of ictal scalp electroencephalography (EEG) in patients with parietal lobe epilepsy (PLE). [file CNS-32-e70713-s004.tif]

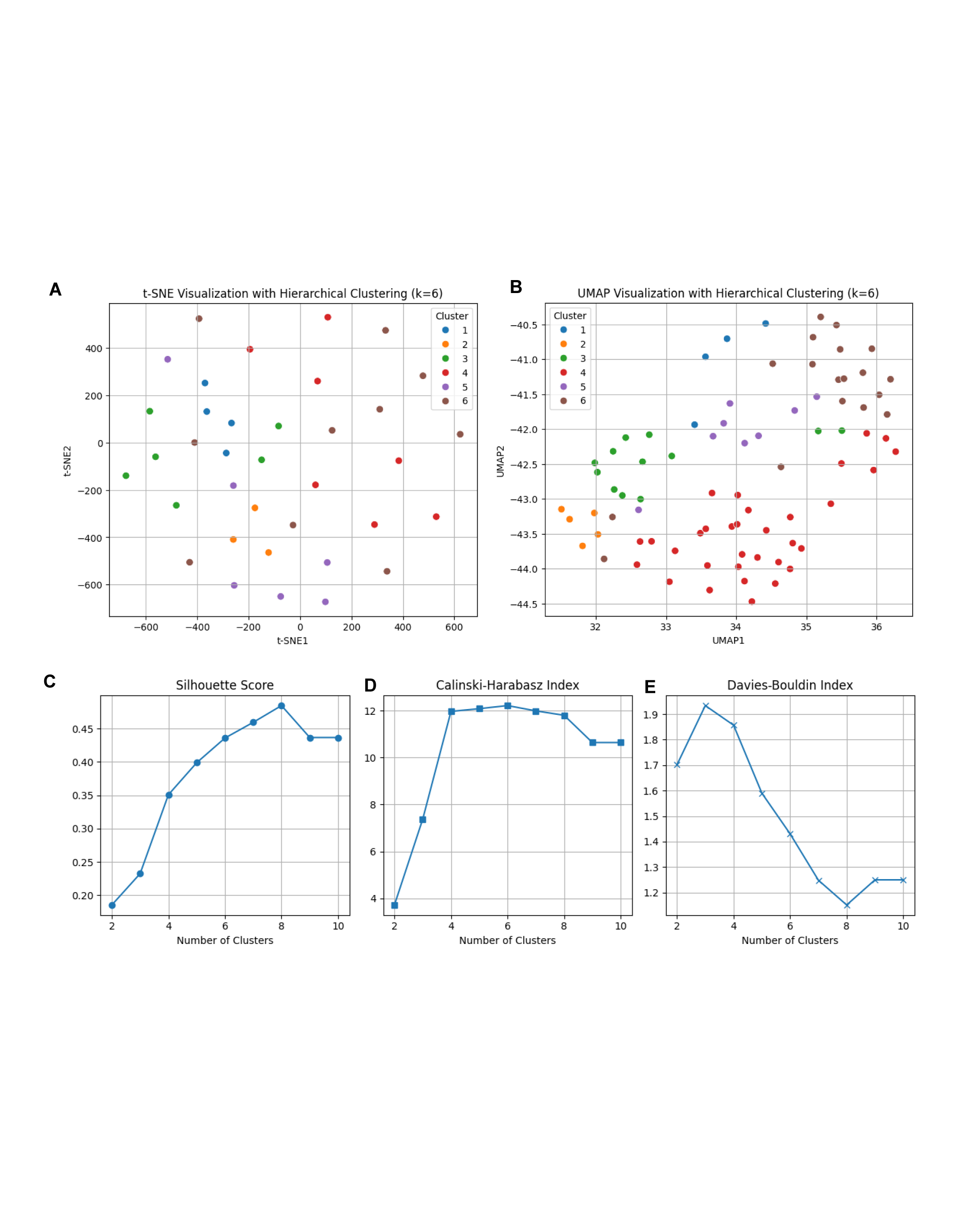

Supplement: Supplementary file 2 — Figure S2: Clustering Validation and Selection of Optimal Cluster Number for Interictal EEG Patterns. [file CNS-32-e70713-s003.tif]

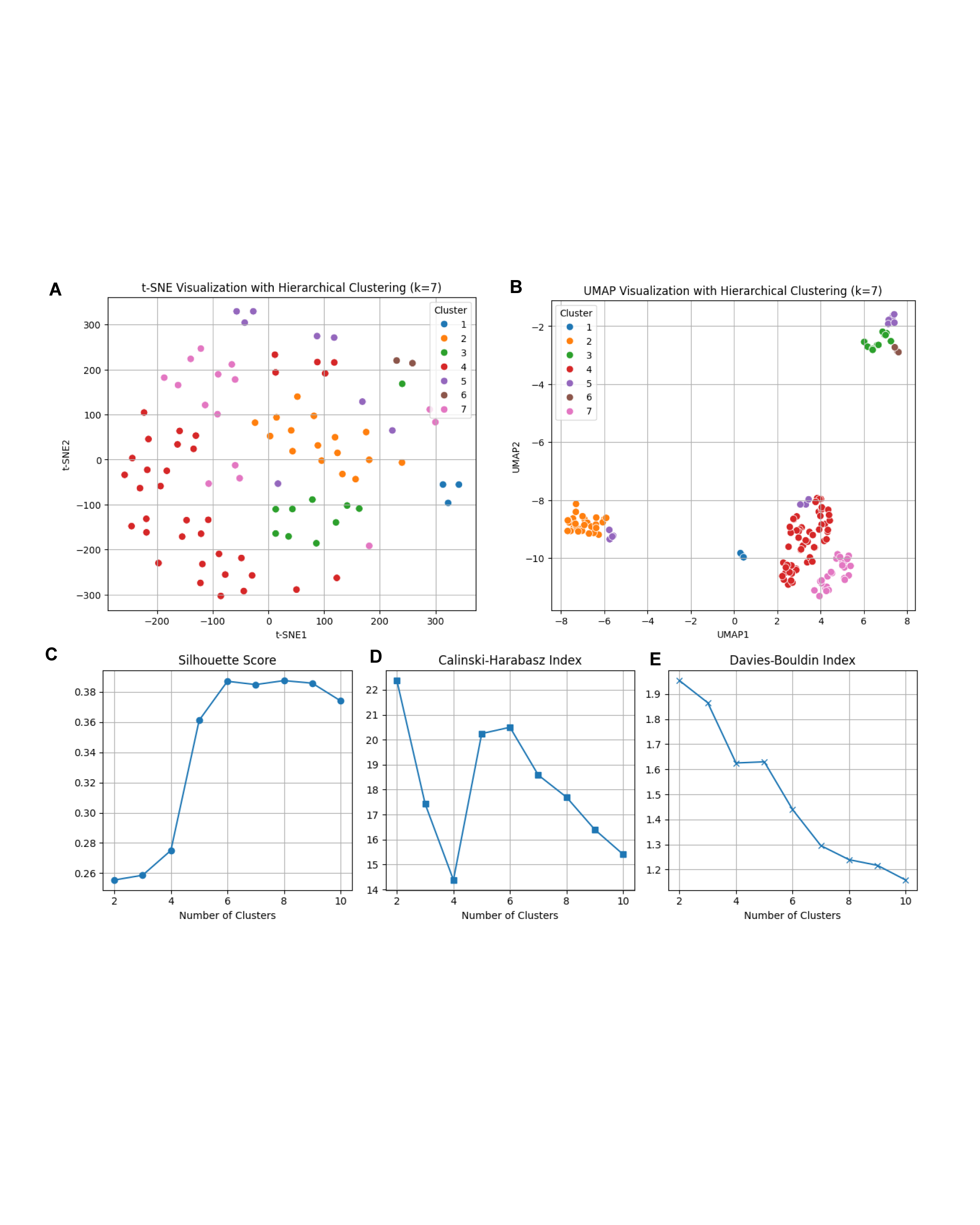

Supplement: Supplementary file 3 — Figure S3: Clustering Validation and Selection of Optimal Cluster Number for Ictal EEG Patterns. [file CNS-32-e70713-s008.tif]

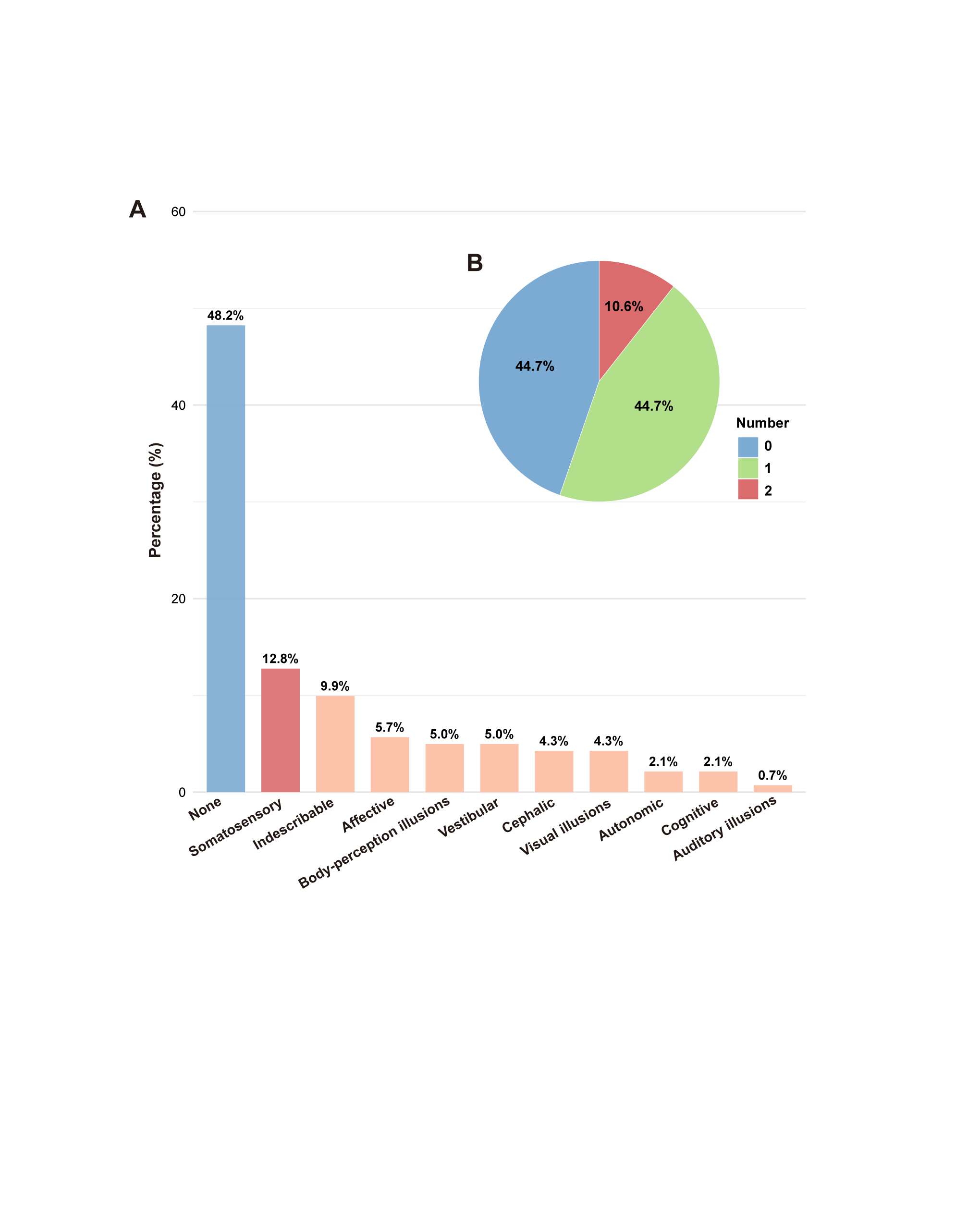

Supplement: Supplementary file 4 — Figure S4: Characteristics of not observable or possibly observable manifestations. [file CNS-32-e70713-s001.tif]

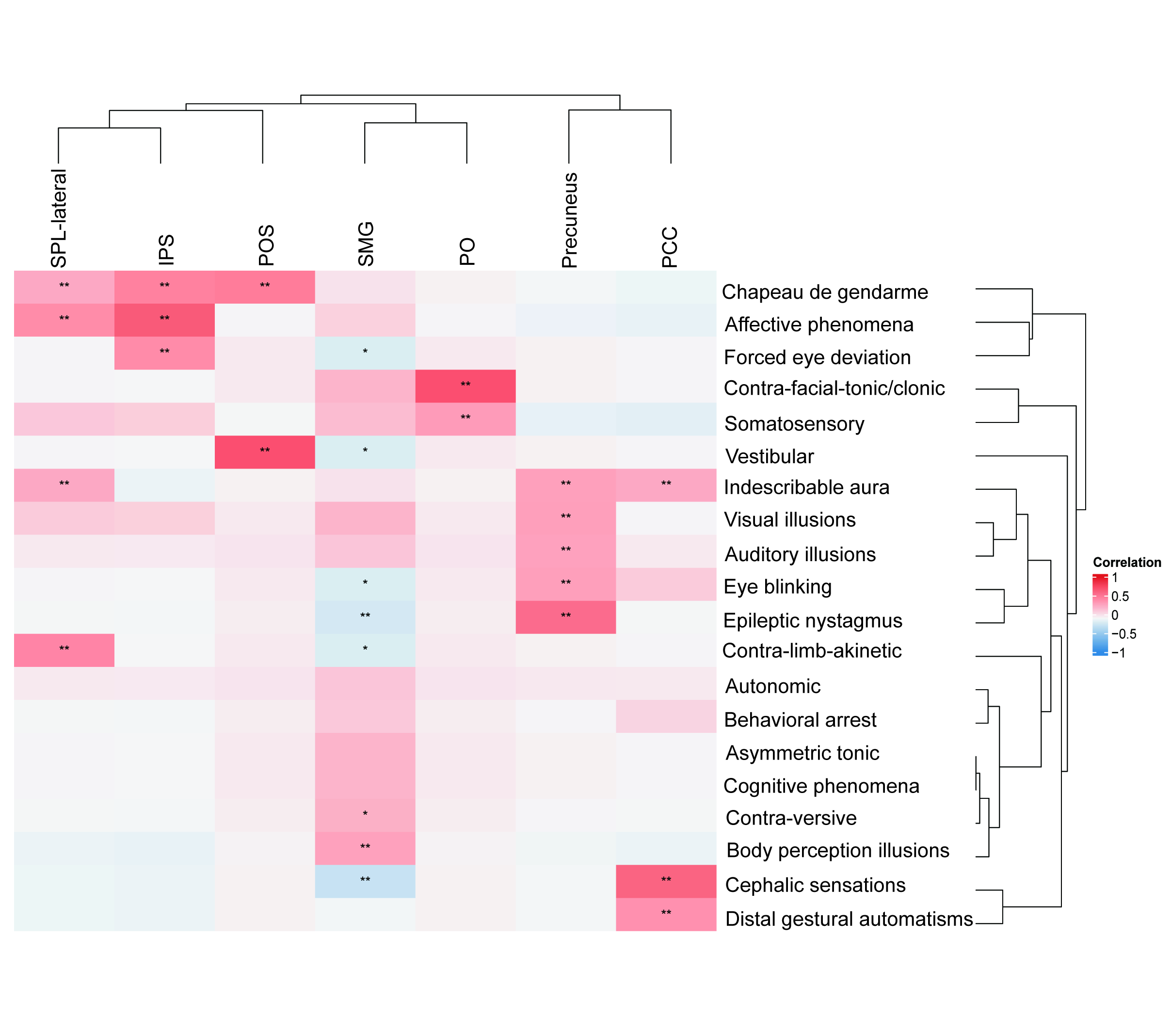

Supplement: Supplementary file 5 — Figure S5: Cluster Heatmap of Pearson's Correlation and Hierarchical Clustering Analyses Between Subgroups and Initial Ictal Semiology with Significant Differences in Patients Undergoing SEEG. [file CNS-32-e70713-s005.tif]
