# Supplementary material for: Anatomo‐Electro‐Clinical Features of Parietal Lobe Epilepsy: Insights From Scalp Video‐Electroencephalography
Source: CNS Neurosci Ther. 2026 Jan 9;32(1):e70713. doi: 10.1002/cns.70713 (PMC12784376; doi:10.1002/cns.70713)
Supplement: Supplementary file 6 — Data S1: cns70713‐sup‐0006‐DataS1.docx. [file CNS-32-e70713-s006.docx]

**Supplementary Figure 1. Proportion of correct localization and lateralization for different morphological types of ictal scalp electroencephalography (EEG) in patients with parietal lobe epilepsy (PLE)**

LVFA = low-voltage fast activity.

**Supplementary Figure 2. Clustering Validation and Selection of Optimal Cluster Number for Interictal EEG Patterns**

(A) t-SNE visualization of clustering results; (B) UMAP visualization of clustering results; (C) Silhouette Score assessing cluster cohesion and separation; (D) Calinski–Harabasz index determining the optimal number of clusters; (E) Davies–Bouldin Index evaluating cluster compactness and separation.

EEG, electroencephalography; t-SNE, t-distributed stochastic neighbor embedding; UMAP, Uniform Manifold Approximation and Projection.

**Supplementary Figure 3. Clustering Validation and Selection of Optimal Cluster Number for Ictal EEG Patterns**

(A) t-SNE visualization of clustering results; (B) UMAP visualization of clustering results; (C) Silhouette Score assessing cluster cohesion and separation; (D) Calinski–Harabasz index determining the optimal number of clusters; (E) Davies–Bouldin Index evaluating cluster compactness and separation.

EEG, electroencephalography; t-SNE, t-distributed stochastic neighbor embedding; UMAP, Uniform Manifold Approximation and Projection.

**Supplementary Figure 4. Characteristics of not observable or possibly observable manifestations**

A. Bar chart representing the percentage of different non-observable or possibly observable manifestations across all seizures. B. Pie chart displaying the distribution of patients according to the number of not observable or possibly observable manifestations experienced (0, 1, or 2).

**Supplementary Figure 5. Cluster Heatmap of Pearson’s Correlation and Hierarchical Clustering Analyses Between Subgroups and Initial Ictal Semiology with Significant Differences in Patients Undergoing SEEG**

The horizontal axis represents different brain subgroups, and the vertical axis represents various initial ictal semiological features. The center of the figure displays a Pearson’s correlation-based clustering heatmap, where color intensity reflects the strength and direction of the correlation: red for positive and blue for negative associations. Both rows and columns were hierarchically clustered to highlight patterns of association. Asterisks denote statistical significance: * P < 0.1 and ** P < 0.05.

PO, parietal operculum; SPL, superior parietal lobule; SMG, supramarginal gyrus; IPS, intraparietal sulcus; POS, parieto-occipital sulcus; PCC, posterior cingulate cortex; Contra, contralateral; IPL, inferior parietal lobule
