# Supplementary material for: Anatomo‐Electro‐Clinical Features of Parietal Lobe Epilepsy: Insights From Scalp Video‐Electroencephalography
Source: CNS Neurosci Ther. 2026 Jan 9;32(1):e70713. doi: 10.1002/cns.70713 (PMC12784376; doi:10.1002/cns.70713)
Supplement: Supplementary file 7 — Table S1: Comparison of epileptogenic parietal subgroups between men and women patients with PLE. Table S2: Comparison of EEG interictal distribution between men and women patients with PLE. Table S3: Comparison of EEG ictal patterns between men and women patients with PLE. Table S4: Comparison of initial ictal semiology between men and women patients with PLE. Table S5: SEEG‐Implanted Patients: Clinical, Sampling, and Scalp EEG Onset Data (n = 28). Table S6: Clinical and Demographic Data for Non‐SEEG Patients (n = 19). [file CNS-32-e70713-s007.docx]

**Supplementary Table 1. Comparison of epileptogenic parietal subgroups between men and women patients with PLE**

| **Characteristics** | **Men (n = 33)** | **Women (n = 14)** | **P value** |
| --- | --- | --- | --- |
| **Surgery location, n (%)** |  |  | 0.649 |
| SPL-lateral | 5 (15.2) | 2 (14.3) |  |
| IPS | 3 (9.1) | 0 |  |
| POS | 1 (3) | 1 (7.1) |  |
| IPL | 12 (36.4) | 7 (50) |  |
| PCC | 2 (6.1) | 2 (14.3) |  |
| PO | 1 (3) | 1 (7.1) |  |
| Precuneus | 3 (9.1) | 0 |  |
| Multisublobar | 6 (18.2) | 1 (7.1) |  |

PLE, parietal lobe epilepsy; SPL, superior parietal lobule; IPS, intraparietal sulcus; POS, parieto-occipital sulcus; IPL, inferior parietal lobule; PCC, posterior cingulate cortex; PO, parietal operculum.

**Supplementary Table 2. Comparison of EEG interictal distribution between men and women patients with PLE**

| **Characteristics** | **Men (n = 44)** | **Women (n = 35)** | **P value** |
| --- | --- | --- | --- |
| **Interictal distribution, n (%)** |  |  | 0.545 |
| Bilateral frontal | 1 (2.3) | 0 |  |
| Bilateral frontotemporal | 1 (2.3) | 3 (8.6) |  |
| Contralateral centroparietal | 3 (6.8) | 0 |  |
| Contralateral frontal | 0 | 1 (2.9) |  |
| Contralateral frontotemporal | 2 (4.5) | 1 (2.9) |  |
| Contralateral posterior | 1 (2.3) | 0 |  |
| Ipsilateral centroparietal | 9 (20.5) | 11 (31.4) |  |
| Ipsilateral frontal | 3 (6.8) | 3 (8.6) |  |
| Ipsilateral frontotemporal | 11 (25) | 5 (14.3) |  |
| No abnormality | 2 (4.5) | 4 (11.4) |  |
| Ipsilateral posterior | 8 (18.2) | 5 (14.3) |  |
| Ipsilateral temporal | 3 (6.8) | 2 (5.7) |  |

**Supplementary Table 3. Comparison of EEG ictal patterns between men and women patients with PLE**

| **Characteristics** | **Men (n = 79)** | **Women (n = 62)** | **P value** |
| --- | --- | --- | --- |
| **Ictal distribution, n (%)** |  |  | 0.588 |
| Bilateral centroparietal | 3 (3.8) | 1 (1.6) |  |
| Bilateral frontal | 3 (3.8) | 5 (7.9) |  |
| Bilateral posterior | 3 (3.8) | 0 |  |
| Contralateral centroparietal | 1 (1.3) | 2 (3.2) |  |
| Contralateral frontal | 2 (2.6) | 0 |  |
| Contralateral posterior | 2 (2.6) | 0 |  |
| Contralateral temporal | 0 | 2 (3.2) |  |
| Ipsilateral centroparietal | 27 (34.6) | 21 (33.3) |  |
| Ipsilateral frontal | 9 (11.5) | 6 (9.5) |  |
| Ipsilateral frontotemporal | 7 (9) | 7 (11.1) |  |
| Midline | 2 (2.6) | 1 (1.6) |  |
| No abnormality | 3 (3.8) | 1 (1.6) |  |
| Ipsilateral posterior | 10 (12.8) | 13 (20.6) |  |
| Ipsilateral temporal | 6 (7.7) | 4 (6.3) |  |
| **Ictal morphology, n (%)** |  |  | 0.546 |
| Low-voltage fast activity | 35 (44.9) | 28 (44.4) |  |
| Spikes or sharp waves | 21 (26.9) | 18 (28.6) |  |
| Obscured | 3 (3.8) | 1 (1.6) |  |
| Slow waves | 19 (24.4) | 16 (25.4) |  |

**Supplementary Table 4. Comparison of initial ictal semiology between men and women patients with PLE**

| **Characteristics** | **Men (n = 99)** | **Women (n = 42)** | **P value** |
| --- | --- | --- | --- |
| **Initial ictal semiology, n (%)** |  |  | 0.135 |
| Affective phenomena | 6 (6.1) | 2 (4.8) |  |
| Contralateral limb akinetic | 3 (3) | 1 (2.4) |  |
| Auditory illusions | 3 (3) | 1 (2.4) |  |
| Autonomic phenomena | 3 (3) | 0 |  |
| Behavioral arrest | 4 (4) | 4 (9.5) |  |
| Bilateral tonic | 3 (3) | 2 (4.8) |  |
| Body perception illusions | 3 (3) | 2 (4.8) |  |
| Cephalic aura | 4 (4) | 0 |  |
| Chapeau de gendarme | 3 (3) | 0 |  |
| Cognitive phenomena | 2 (2) | 3 (7.1) |  |
| Contralateral facial tonic/clonic | 6 (6.1) | 3 (7.1) |  |
| Contralateral limb tonic/clonic | 0 | 2 (4.8) |  |
| Contralateral versive | 8 (8.1) | 1 (2.4) |  |
| Epileptic nystagmus | 4 (4) | 0 |  |
| Eye blinking | 5 (5.1) | 1 (2.4) |  |
| Forced eye deviation | 3 (3) | 0 |  |
| Gestural automatisms-distal | 6 (6.1) | 4 (9.5) |  |
| Hyperkinetic behavior | 3 (3) | 0 |  |
| Indescribable aura | 7 (7.1) | 8 (19) |  |
| Somatosensory | 16 (16.2) | 2 (4.8) |  |
| Vestibular | 4 (4) | 3 (7.1) |  |
| Visual illusions | 3 (3) | 3 (7.1) |  |

**Supplementary Table 5. SEEG-Implanted Patients: Clinical, Sampling, and Scalp EEG Onset Data (n = 28)**

| Patients | M/F | Age at onset | MRI | Histopathological types | SEEG explored | SOZ/Surgical intervention site | Scalp EEG ictal onset distribution | Scalp EEG ictal onset morphology |
| --- | --- | --- | --- | --- | --- | --- | --- | --- |
| PLE1 | F | 13 | Negative | Not available | MCC, PoCG, AG, precuneus, PCC, O, POS, SMG, T | SMG | Posterior | LVFA |
| PLE10 | M | 11 | Positive | Gangliocytoma | PO, Ins, T, SMG, AG, PCC | SMG | Frontal | Slow |
| PLE11 | M | 24 | Positive | Glioma | SPL-lateral, PCC, SMG, T, O | PCC | Frontotemporal | Spike or sharp waves |
| PLE12 | M | 6 | Negative | FCD | AG, PCC, SMG, T, PO, Ins, F, ACC, MCC | SMG | Centroparietal | LVFA |
| PLE13 | M | 11 | Positive | Nonspecific | PoCG, PO, PCC, SMG, precuneus, SPL-lateral, PCG | PO | Centroparietal | LVFA |
| PLE14 | F | 13 | Positive | Not available | SPL-lateral, precuneus, SMG, PoCG, MCC, Ins, F | SMG | Bilateral _frontal | Slow |
| PLE15 | F | 8 | Positive | Gliosis | SPL-lateral, precuneus, SMG, PoCG, MCC, Ins, F | SMG | Frontotemporal | Slow |
|  |  |  |  |  |  |  | Posterior | Slow |
| PLE16 | M | 6 | Positive | Gliosis | MCC, T, SMG, precuneus, O, AG, PCC, PoCG | PCC | Contralateral_frontal | Slow |
| PLE19 | M | 14 | Positive | Gliosis | ACC, MCC, PCC, Ins, F, PoCG, SMG | SMG | Centroparietal | LVFA |
| PLE2 | F | 10 | Negative | FCD | SPL-lateral, PCC, SMG, MCC, AG, T, PO, Ins | PCC | Contralateral_temporal | Spike or sharp waves |
|  |  |  |  |  |  |  | Bilateral_centroparietal | Spike or sharp waves |
|  |  |  |  |  |  |  | Frontotemporal | LVFA |
| PLE21 | M | 19 | Negative | FCD | T, PO, Ins, SMG, AG, PCC | SMG | Centroparietal | LVFA |
|  |  |  |  |  |  |  | Frontal | Slow |
| PLE26 | F | 10 | Positive | Not available | SPL-lateral, precuneus, SMG, PoCG, MCC, Ins, F | SMG | Centroparietal | Spike or sharp waves |
|  |  |  |  |  |  |  | Bilateral_frontotemporal | Spike or sharp waves |
| PLE31 | M | 31 | Negative | FCD | F, ACC, T, precuneus, O, AG | POS | Frontal | Slow |
|  |  |  |  |  |  |  | Contralateral_posterior | LVFA |
| PLE32 | M | 5 | Positive | FCD | F, PCG, MCC, PoCG, SPL-lateral, precuneus, O, T, POS | Precuneus | Bilateral_centroparietal | LVFA |
|  |  |  |  |  |  |  | Frontal | LVFA |
| PLE33 | M | 7 | Positive | Nonspecific | PCG, MCC, PoCG, SMG, PCC, AG, precuneus, SPL-lateral, T | Precuneus | Bilateral_centroparietal | LVFA |
|  |  |  |  |  |  |  | Bilateral_posterior | Spike or sharp waves |
| PLE34 | M | 7 | Positive | FCD | IPS, PCC, precuneus, POS, O, SMG, AG, T | POS | Posterior | LVFA |
| PLE35 | M | 15 | Positive | Not available | PoCG, MCC, SPL-lateral, precuneus, PCC, AG | SPL-lateral | Centroparietal | LVFA |
| PLE37 | M | 5 | Positive | FCD | PoCG, MCC, SPL-lateral, SMG, precuneus, PCC, AG, Ins | IPS | Temporal | Spike or sharp waves |
| PLE38 | M | 1 | Positive | Nonspecific | PCC, SPL-lateral, SMG, precuneus, POS, PoCG | IPS | Frontal | Slow |
| PLE39 | F | 17 | Positive | FCD | T, SMG, PCC, O, Ins, F, MCC | SPL-lateral, SMG | Frontotemporal | Spike or sharp waves |
| PLE42 | M | 11 | Positive | FCD | PCG, MCC, PO, SMG, PCC, AG, T, O, precuneus | SPL-lateral, SMG | Frontotemporal | Spike or sharp waves |
| PLE6 | M | 11 | Positive | FCD | PCG, PoCG, precuneus, SMG, PCC, MCC, Ins, F, T | SPL-lateral, precuneus, PCC, SMG | Posterior | LVFA |
| PLE8 | M | 13 | Positive | FCD | Ins, F, SMG, precuneus, O, T | SMG | No abnormality | Obscured |
| PLE9 | F | 6 | Negative | FCD | PoCG, MCC, SPL-lateral, precuneus, AG, SMG, PCC, T | SPL-lateral, precuneus | Midline | Spike or sharp waves |
| PLE51 | F | 9 | Negative | FCD | T, AG, PCC, SMG, PCG, PoCG, Ins | SMG | Posterior | Spike or sharp waves |
| PLE52 | M | 18 | Positive | FCD | SPL-lateral, precuneus, PCC, SMG, T, PoCG, Ins, T | SPL-lateral, precuneus, PCC | Posterior | Slow |
| PLE53 | F | 11 | Negative | FCD | SPL-lateral, PCC, SMG, MCC, AG, T, PO, Ins | PCC | Frontotemporal | LVFA |
|  |  |  |  |  |  |  | Contralateral_centroparietal | LVFA |
| PLE22 | M | 13 | Positive | FCD | Ins, F, SMG, precuneus, O, T, SPL-lateral, PCG | SMG, PO | Midline | Spike or sharp waves |

PLE, parietal lobe epilepsy; M/F, male/female; SEEG, stereoelectroencephalography; SOZ, seizure onset zone; EEG, electroencephalography; MCC, middle cingulate cortex; PoCG, postcentral gyrus; AG, angular gyrus; PCC, posterior cingulate cortex; O, occipital lobe; POS, parieto-occipital sulcus; SMG, supramarginal gyrus; T, temporal lobe; F, frontal lobe; PO, parietal operculum; Ins, insular lobe; SPL-lateral, lateral superior parietal lobule; ACC, anterior cingulate cortex; PCG, precentral gyrus; IPS, intraparietal sulcus; LVF, low-voltage fast activity; MRI, magnetic resonance imaging.

**Supplementary Table 6. Clinical and Demographic Data for Non-SEEG Patients (n = 19)**

| Patients | M/F | Age at onset | MRI | Surgical intervention site |
| --- | --- | --- | --- | --- |
| PLE17 | M | 10 | Negative | SMG |
| PLE18 | M | 8 | Positive | SMG |
| PLE20 | M | 10 | Positive | SMG |
| PLE23 | M | 8 | Negative | SMG |
| PLE24 | F | 28 | Positive | SMG |
| PLE30 | M | 7 | Positive | SPL-lateral, AG, POS, IPS |
| PLE40 | M | 3 | Positive | SPL-lateral |
| PLE41 | F | 6 | Positive | SPL-lateral |
| PLE4 | M | 7 | Positive | SPL-lateral |
| PLE5 | M | 1 | Positive | SPL-lateral |
| PLE7 | F | 11 | Negative | PO |
| PLE43 | M | 4 | Negative | SPL-lateral |
| PLE44 | F | 5 | Positive | Precuneus |
| PLE45 | M | 30 | Positive | AG |
| PLE46 | F | 8 | Negative | SPL-lateral |
| PLE47 | M | 6 | Positive | SMG |
| PLE48 | M | 15 | Positive | IPS |
| PLE49 | M | 16 | Negative | SMG |
| PLE50 | M | 12 | Positive | SMG |

PLE, parietal lobe epilepsy; M/F, male/female; AG, angular gyrus; POS, parieto-occipital sulcus; SMG, supramarginal gyrus; PO, parietal operculum; SPL-lateral, lateral superior parietal lobule; IPS, intraparietal sulcus; MRI, magnetic resonance imaging.
