# Supplementary material for: Anatomo‐Electro‐Clinical Features of Parietal Lobe Epilepsy: Insights From Scalp Video‐Electroencephalography
Source: CNS Neurosci Ther. 2026 Jan 9;32(1):e70713. doi: 10.1002/cns.70713 (PMC12784376; doi:10.1002/cns.70713)
Supplement: Supplementary file 8 — Data S2: cns70713‐sup‐0008‐DataS2.docx. [file CNS-32-e70713-s002.docx]

**Supplementary****Text 1. Ictal and Interictal Scalp EEG Analysis**

The distribution of EEG was analyzed based on electrode site and lateralization of the recorded electrical activity. None of the included patients exhibited more than three ictal EEG patterns. We analyzed a minimum of three seizures per patient, with each seizure considered a separate ictal EEG pattern. If a patient experienced more than three seizures with distinct ictal onset patterns were selected for analysis to avoid overrepresentation. Every distinct interictal pattern recorded during monitoring was analyzed, and no patient had more than four interictal EEG patterns included in the analysis.

The definition of ictal onset morphology was as follows: (1) Low-voltage fast activity: (a) sudden disappearance of frequent interictal discharges followed by voltage suppression; (b) voltage suppression or flattening (amplitude <10 μV); and (c) low-voltage fast activity with frequency ≥13 Hz and amplitude <25 μV; (2) Obscured, if the EEG ictal onset was blurred by muscle artifacts; (3) Slow waves; (4) Spikes or sharp waves.

The scalp EEG distribution was categorized as frontal (Fp1, Fp2, F3, F4, Fz, F7, F8), frontotemporal (Fp1, Fp2, F3, F4, Fz, F7, F8, T3, T4), temporal (F7, F8, T3, T4, T5, T6), centroparietal (C3, C4, P3, P4, Cz, Pz), and posterior (P3, P4, O1, O2, T5, T6, Pz).

**Supplementary Text 2. Clustering Validation and Selection of Optimal Cluster Number for Interictal and Ictal EEG Patterns**

Clustering Selection Process for Interictal EEG patterns: The choice of six clusters was supported by visual and quantitative assessments. t-SNE visualization of hierarchical clustering (k = 6) showed clear separation between clusters, with each group distinctly colored and well defined in 2D space, demonstrating strong intra-cluster similarity and inter-cluster separation. UMAP results further corroborated this, revealing similar clear separation with minimal overlap. Although the Silhouette Score was highest for eight clusters, indicating optimal cohesion and separation, the Calinski–Harabasz Index favored six clusters, suggesting the best balance between inter- and intra-cluster variance. In contrast, the Davies–Bouldin Index was lowest for eight clusters, indicating minimal similarity between clusters. Despite eight clusters performing best in some metrics, the combination of visual clarity and the overall evaluation indices led us to select six clusters as the more practical and reliable choice.

Clustering Selection Process for Ictal EEG patterns: The choice of seven clusters was supported by visual and quantitative assessments. t-SNE visualization of hierarchical clustering (k = 7) showed clear separation between clusters, with each group distinctly colored and well defined in 2D space, indicating strong intra-cluster similarity and inter-cluster separation. UMAP results further corroborated this, revealing similar clear separation and minimal overlap. Although the Silhouette Score was highest for eight clusters, indicating optimal cohesion and separation, the score for seven clusters was also close to its maximum, demonstrating a good clustering effect. For the Calinski–Harabasz Index, the highest value was observed for six clusters, but seven clusters still performed well, indicating a favorable balance between inter- and intra-cluster variance. Although the Davies–Bouldin Index was lowest for 10 clusters, suggesting minimal similarity between clusters, selecting 10 clusters could result in over-partitioning and loss of practical interpretability. Considering the visual results together with other evaluation metrics, seven clusters emerged as the more practical and reliable choice.

**Supplementary Text 3. Scalp EEG Features across PLE Subgroups**

Hierarchical cluster analysis of interictal discharges distribution in scalp EEG and involved brain areas of the 79 analyzed interictal EEG samples identified six major clusters (Figures 3A and 4). The first cluster was associated with the precuneus, parieto-occipital sulcus (POS), and interictal discharges on contralateral centroparietal, contralateral posterior, and ipsilateral posterior electrodes. The second cluster was linked to the intraparietal sulcus (IPS) and interictal discharges on ipsilateral temporal electrodes. The third cluster was associated with multisublobar regions and interictal discharges on contralateral frontal and contralateral frontotemporal electrodes. The fourth cluster was associated with the posterior cingulate cortex (PCC) and interictal discharges on bilateral frontal and ipsilateral frontotemporal electrodes. The fifth cluster was associated with the lateral superior parietal lobule (SPL-lateral), parietal operculum, and interictal discharges on bilateral frontotemporal and ipsilateral centroparietal electrodes. The sixth cluster was associated with the inferior parietal lobule (IPL), no abnormality on interictal EEG, and interictal discharges on ipsilateral frontal electrodes. In addition, in the fifth cluster, smaller sub-clusters at a lower level grouped interictal discharges on centroparietal electrodes with SPL-lateral, separating them from the parietal operculum and interictal discharges on bilateral frontal-temporal electrodes.

Hierarchical cluster analysis of ictal onset patterns in scalp EEG and involved brain areas of the 141 analyzed seizures identified six major clusters (Figures 3B and 4). The first cluster was associated with IPL, IPS, slow-wave onset, and ictal discharges on bilateral frontal, ipsilateral frontal, and ipsilateral temporal electrodes. The second cluster was associated with POS and ictal discharges on contralateral and ipsilateral posterior electrodes. The third cluster was associated with the precuneus, spikes or sharp-wave onset, and ictal discharges on bilateral posterior and bilateral centroparietal electrodes. The fourth cluster was associated with multisublobar regions and ictal discharges on midline electrodes. The fifth cluster was associated with PCC and ictal discharges on contralateral centroparietal, contralateral frontal, ipsilateral frontotemporal, and contralateral temporal electrodes. The sixth cluster was associated with SPL-lateral, parietal operculum, low-voltage fast activity (LVFA) onset, and ictal discharges on ipsilateral centroparietal electrodes. In addition, in the sixth cluster, smaller sub-clusters at a lower level grouped ictal discharges on centroparietal electrodes, LVFA onset, and SPL-lateral together, separating them from the parietal operculum. Moreover, ictal onset classified as “obscured” was treated as equivalent to “no abnormality” and thus grouped into one category.

**Supplementary Text 4. Analysis of Sex/Gender Effects**

Supplementary analyses were performed to assess the influence of sex/gender on interictal EEG patterns (distribution), ictal EEG patterns (distribution and morphology), initial ictal semiology, and the parietal subgroups involved in the epileptogenic zone. No significant differences were observed between men and women patients across these measures (see Supplementary Tables 1–4). Consequently, sex/gender was not included as a variable in the clustering analysis.
